# A possible association between the -2518 A>G *MCP-1* polymorphism and insulin resistance in school children

**DOI:** 10.20945/2359-3997000000012

**Published:** 2018-01-01

**Authors:** Inés Matia-García, Lorenzo Salgado-Goytia, Luz Elena Ramos-Arellano, José Francisco Muñoz-Valle, Adakatia Armenta-Solís, Olga Lilia Garibay-Cerdenares, Mónica Ramírez, Isela Parra-Rojas

**Affiliations:** 1 Universidad Autónoma de Guerrero Universidad Autónoma de Guerrero Facultad de Ciencias Químico Biológicas Chilpancingo Guerrero México Facultad de Ciencias Químico Biológicas, Universidad Autónoma de Guerrero, Chilpancingo, Guerrero, México; 2 Universidad de Guadalajara Universidad de Guadalajara Instituto de Investigación en Ciencias Biomédicas Departamento de Biología Molecular y Genómica, Centro Universitario de Ciencias de la Salud Jalisco México Instituto de Investigación en Ciencias Biomédicas, Departamento de Biología Molecular y Genómica, Centro Universitario de Ciencias de la Salud, Universidad de Guadalajara, Jalisco, México; 3 Universidad Autónoma de Guerrero Universidad Autónoma de Guerrero Facultad de Medicina Acapulco Guerrero México Facultad de Medicina, Universidad Autónoma de Guerrero, Acapulco, Guerrero, México; 4 Universidad Autónoma de Guerrero Universidad Autónoma de Guerrero Facultad de Ciencias Químico Biológicas Chilpancingo Guerrero México Cátedra CONACyT, Facultad de Ciencias Químico Biológicas, Universidad Autónoma de Guerrero, Chilpancingo, Guerrero, México; 5 Universidad Autónoma de Guerrero Universidad Autónoma de Guerrero Facultad de Ciencias Químico Biológicas Laboratorio de Investigación en Obesidad y Diabetes Ciudad Universitaria Guerrero México Laboratorio de Investigación en Obesidad y Diabetes, Facultad de Ciencias Químico Biológicas, Universidad Autónoma de Guerrero, Ciudad Universitaria, Guerrero, México

**Keywords:** Insulin resistance, MCP-1, polymorphism, obesity, children

## Abstract

**Objective:**

Monocyte chemoattractant protein 1 (MCP-1) has been suggested to be involved in the pathophysiology of insulin resistance (IR); therefore, variants in the *MCP-1* gene may contribute to the development of this disease. The aim of this study was to analyze the relationship of the -2518 A>G *MCP-1* (rs1024611) gene polymorphism with insulin resistance in Mexican children.

**Subjects and methods:**

A cross-sectional study was performed in 174 children, including 117 children without insulin resistance and 57 children with IR, with an age range of 6-11 years. Levels for serum insulin and high-sensitivity C-reactive protein were determined. The -2518 A>G *MCP-1* polymorphism was identified by the polymerase chain reaction-restriction fragment length polymorphism method. Insulin resistance was defined as a HOMA-IR in the upper 75^th^ percentile, which was ≥ 2.4 for all children.

**Results:**

Genotype frequencies of the rs1024611 polymorphism for the insulin-sensitive group were 17% AA, 48% AG and 35% GG, and the frequency of G allele was 59%, whereas frequencies for the insulin-resistant group were 12% AA, 37% AG and 51% GG, and the frequency of G allele was 69%. The genotype and allele frequencies between groups did not show significant differences. However, the GG genotype was the most frequent in children with IR. The GG genotype was associated with insulin resistance (OR = 2.2, *P* = 0.03) in a genetic model.

**Conclusion:**

The -2518 A>G *MCP-1* gene polymorphism may be related to the development of insulin resistance in Mexican children.

## INTRODUCTION

The marked increase in pediatric obesity in the past decade has resulted in unprecedented increases in the incidence of type 2 diabetes mellitus among children and adolescents (1,2). There is substantial evidence that obesity is the main determinant of insulin resistance in children and that it increases the risk not only for the metabolic syndrome in adulthood but also for cardiovascular disease and type 2 diabetes later in life (3,4).

Obesity is associated with a chronic low-grade inflammatory state, characterized by enhanced production of multiple cytokines and chemokines. Monocyte chemoattractant protein-1 (MCP-1) is a chemokine produced by adipose tissue and other tissues. The production of MCP-1 in obesity is triggered when adipocytes are exposed to inflammatory cytokines and fatty acids. Other cells that produce MCP-1 in obesity include hepatocytes, skeletal muscle cells, monocytes, vascular smooth muscle and endothelial cells. MCP-1 production results in initiation and propagation of the inflammatory response in obesity (5,6). Some studies have found that plasma levels of MCP-1 are increased in obese adults (7) and in obese children (8) compared to lean controls. Furthermore, MCP-1 signaling has a direct role in the development of obesity. Younce and cols. reported that MCP-1-induced adipogenesis in 3T3-L1 cells is independent of PPAR gamma activation (9). Mice with CCR2 deficiency had attenuated deposition of visceral fat and insulin resistance when challenged with a high fat diet (10). Moreover, *MCP-1* had an angiogenic effect on endothelial cells (11); therefore, it can contribute to the expansion and remodeling of adipose tissue (12).

In diabetic subjects, the high glucose-induced inflammatory process is characterized by the cooperation of a complex network of inflammatory molecules, such as cytokines, adhesion molecules, growth factors, and chemokines. The high glucose concentration induces an increase in the synthesis and release of MCP-1 by endothelial cells (EC) and smooth muscle cells (SMC) (13). MCP-1 is a potent chemotactic factor that regulates monocyte and macrophage migration and infiltration at sites of inflammation (14). The interaction of MCP-1 with its receptor CCR2 is considered pivotal in obesity-induced insulin resistance. Several groups have reported that mice with targeted deletions in the genes for *MCP-1/CCL2* and its receptor *CCR2* have reduced adipose tissue macrophage (ATM) content, decreased inflammation in fat and protection from high-fat (HF) diet-induced insulin resistance (15). Conversely, mice overexpressing MCP-1 in adipose tissue have an increased number of ATMs along with insulin resistance (16). Therefore, the MCP-1-CCR2 axis is of central importance for promoting ATM recruitment and insulin resistance in mice. Zineh and cols. reported that serum levels of MCP-1 increased in children with type 1 diabetes compared with the control group (17). In another study in adult subjects, serum concentrations of MCP-1 were higher in patients with type 2 diabetes than in normal subjects (18). The glucose and insulin appear to exert effects on MCP-1 secretion, and this interaction might be important for the development of insulin resistance in children (19).

A single nucleotide polymorphism characterized by a chance of A>G at position -2518 in the *MCP-1* distal promoter regulatory region affects MCP-1's transcription activity in response to IL-1β (20). This polymorphism has been associated with the development of chronic diseases such as obesity, hypertension, atherosclerosis, type 2 diabetes and insulin resistance (21-23). Briefly, in a German population, the *MCP-1* -2518G allele was associated with decreased prevalence of insulin resistance and type 2 diabetes (24). Another study carried out in a Japanese population found that in obese diabetics, -2518AA carriers had increased insulin resistance compared to -2518GG carriers (23). Alternatively, in an adult population of western Mexico, it was found that subjects without insulin resistance presented the -2518A allele more frequently than their counterparts with IR (25). In light of these contradictory results, it is important to perform replication studies in different populations to determine the association of this polymorphism with insulin resistance. In the Mexican population, the distributions of genotypic frequencies of this polymorphism in adults and its relations with bladder cancer and tuberculosis have been reported (26,27). Genotypic frequencies and their possible relationship to insulin resistance have not been identified in children. The aim of this study was to investigate whether the -2518 A>G *MCP-1* polymorphism is associated with insulin resistance in Mexican children.

## SUBJECTS AND METHODS

### Subjects

We analyzed a total of 174 unrelated children (86 girls and 88 boys), with an age range of 6-11 years, who were divided into two groups: 117 children without IR and 57 children with IR. The participants were all born in the State of Guerrero, Mexico, with a family history of ancestors, at least back to the third generation, born in this state. All children with evidence of infectious disease or with any treatment that could influence biochemical or hematological parameters were excluded from the study. Informed written consent was obtained from all parents or guardians before enrollment of children in the study according to the ethical guidelines of the 2008 Declaration of Helsinki. Approval for the study was obtained from the Research Ethics Committee of the University of Guerrero.

### Clinical and anthropometric measurements

Body weight was determined in light clothes and without shoes using a body composition monitor (Tanita BC-553, Arlington, USA). Height and body circumferences were measured with a stadiometer and anthropometric tape (Seca, Hamburg, Germany), respectively. The classification of obesity was made using the 2000 Centers for Disease Control and Prevention growth charts, with normal weight defined as the 5^th^-85^th^ percentiles and obesity as ≥ 95^th^ percentile (28). The four skinfold thicknesses (triceps, biceps, subscapular and suprailiac) were measured with a plicometer (Dynatronics Co, Salt Lake City, USA) and blood pressure (BP) with an aneroid sphygmomanometer (Riester CE 0124, Jungingen, Germany). Hypertension was defined as systolic or diastolic blood pressure in the 95^th^ percentile or higher for age and sex, using an average of 2 measurements (29).

### Laboratory assessment

A fasting blood sample was obtained from each child by antecubital venipuncture. Serum glucose levels were analyzed with semi-automated equipment (COBAS MIRA), and insulin levels were measured using an enzyme-linked immunosorbent assay (GenWay INS-EASIA kit). Intra-assay and inter-assay variation coefficients were 4.8% and 8.1%, respectively, and the detection limit was 0.17 µU/mL. The homeostasis model assessment of insulin resistance was used to determine insulin resistance in children. This score was calculated with the following formula: fasting serum insulin (μU/mL) x fasting plasma glucose (mmol/L)/22.5 (30). Insulin resistance was defined as a HOMA-IR at or above the 75^th^ percentile, which was ≥ 2.4 for all children. Leukocyte and platelet counts were determined using an ADVIA-60 (Bayer Diagnostics). High-sensitivity C-reactive protein (hsCRP) levels were measured by a turbidimetry assay (BioSystems SA) with a sensitivity of 0.06 mg/L and intra-assay and inter-assay variation coefficients of 1.8% and 3.6%, respectively.

### Genotyping analysis

Genomic DNA (gDNA) was extracted from peripheral leukocytes obtained from whole blood samples, according to the Miller method. The analysis of the -2518 A>G *MCP-1* (rs1024611) polymorphism was performed by the polymerase chain reactionrestriction fragment length polymorphism (PCR-RFLP) method, using the following primers: 5′CACAGAGAGAGTCTGGCCACGT3′ (forward) and 5′CCAACAGAGGACTCTTGGTCT3′ (reverse). The reaction was carried out in a final volume of 20 μl, adding buffer 1X to 2.5 mM MgCl_2_, 0.2 mM dNTPs, 0.2 mM of each primer, 2.0 U/μl Taq polymerase (Invitrogen Life Technologies) and 0.1 µg/mL of gDNA. PCR was performed with an initial denaturation at 94 °C for 5 min, followed by 25 cycles of amplification consisting of 94 °C for 30 s of denaturation, 63 °C for 30 s of annealing and 72 °C for 30 s of extension, and a final extension step at 72 °C for 5 min. The amplified product of 234 bp was digested with PvuII restriction enzyme (New England Biolabs) for 2 hours at 37 °C and analyzed by electrophoresis in 6% polyacrylamide gel (Invitrogen™ life technologies) stained with silver nitrate. The AA genotype lacking the PvII site migrated as a 234 bp fragment, whereas the GG genotype was cleaved and appeared as 159 bp and 75 bp fragments.

### Statistical analysis

The analysis was performed with STATA software (V.9.2). Differences in variables between groups were evaluated using a chi-square test for categorical variables, Student's *t* test for continuous variables with symmetrical distributions (data are presented as the mean and standard deviation), and a Mann-Whitney U test for variables without symmetrical distributions (data are presented as median and 5^th^ to 95^th^ percentiles). The genotype and allele frequencies were determined by direct counting, and a chi-square test was used to calculate the Hardy-Weinberg Equilibrium in case and control groups. To evaluate the association between insulin resistance and the polymorphism under investigation, we used a logistic regression model that was adjusted by age, gender, obesity and hypertension. P values < 0.05 were considered significant.

## RESULTS

The study was conducted in a total of 174 children, divided into two groups: 117 children without IR were recruited as a control group, and 57 children with IR were the case group. The descriptive characteristics of the participants are summarized in Table 1. The median age of the children was 9 years, without differences between groups in age or gender. The insulin-resistant children had increases in all measures of central and peripheral adiposity and in both systolic and diastolic blood pressure compared to the group with insulin sensitivity. We found a high prevalence of obesity (73.7%) in the insulin-resistant group compared with the insulin-sensitive group (36.8%). The group with IR also showed an increase in serum hsCRP levels but not in leukocyte or platelet counts.

**Table 1:** Clinical and laboratory variables of the studied groups

Variables		All children (n = 174)	Group without IR (n = 117)	Group with IR (n = 57)	*P* value
Age (years)‡		9 (6-11)	9 (6-11)	9 (6-11)	0.08
Gender % (n)*					0.36
	Male	50.6 (88)	53 (62)	45.6 (26)	
	Female	49.4 (86)	47 (55)	54.4 (31)	
Weight (kg)‡		33.2 (20.1-59.1)	30.7 (19.7-48.8)	44.4 (23-68.2)	< 0.001
Height (cm)†		132.6 ± 11.3	131.1 ± 10.6	137.2 ± 12.1	0.001
BMI (kg/m^2^)‡		18.4 (14.3-27)	17.3 (14.1-24.9)	23.5 (14.9-29.9)	< 0.001
Obesity % (n)*					< 0.001
	No	51.1 (89)	63.2 (74)	26.3 (15)	
	Yes	48.9 (85)	36.8 (43)	73.7 (42)	
Waist circumference (cm)‡		67 (54-88)	66 (53-84)	80.3 (58-96)	< 0.001
Hip circumference (cm)‡		75 (62-96)	73 (62-89)	85 (66-104)	< 0.001
Waist-to-hip ratio‡		0.9 (0.8-0.97)	0.9 (0.8-0.97)	0.9 (0.8-1.0)	0.001
Arm circumference (cm)‡		21 (16-28)	20 (16-26)	24.5 (17-33)	< 0.001
Biceps skinfold (mm)†		15.5 ± 4.8	14.8 ± 4.8	17.6 ± 4.3	< 0.001
Triceps skinfold (mm)‡		15 (8.5-21.5)	13.5 (8-21.5)	18 (10-22)	< 0.001
Subscapular skinfold (mm)‡		13 (6-22)	12 (5.5-21)	18.5 (8-25)	< 0.001
Suprailiac skinfold (mm)†		17.9 ± 5.5	17.0 ± 5.5	20.7 ± 4.7	< 0.001
SBP (mmHg)‡		98 (81-115)	96 (81-110)	101 (80-124)	< 0.001
DBP (mmHg)‡		58 (48-71)	57 (48-68)	60 (44.5-78)	< 0.001
Glucose (mg/dL)‡		96 (74-112)	95 (72-112)	98 (78-114)	0.06
Insulin (µU/mL)‡		6.9 (0.8-22.4)	4.7 (0.6-9.2)	13.7 (9.9-31.9)	< 0.001
HOMA-IR‡		1.2 (0.-4.9)	0.6 (0-2.1)	3.1 (2.5-7.3)	< 0.001
hsCRP (mg/L)‡		0.8 (0.1-6.5)	0.6 (0.1-6.0)	1.6 (0.1-8.3)	0.004
Leukocytes (10^3^/mm^3^)‡		7.7 (5.0-12.3)	7.6 (5.0-12.5)	7.9 (5-11.5)	0.08
Platelets (10^3^/mm^3^)‡		308 (219-413)	306 (219-440)	316 (218-410)	0.22

* Values are expressed as percentages and *n*. Chi-square test.

† Values are expressed as the means ± SD. Student's *t* test.

‡ Values are expressed as median (p5-p95). Mann-Whitney test. IR: insulin resistance; BMI: body mass index; SBP: systolic blood pressure; DBP: diastolic blood pressure; HOMA-IR: homeostasis model of assessment-insulin resistance; hsCRP: high sensitivity C-reactive protein.

Demographic, clinical and metabolic variables were compared between children grouped according to their genotypes of the -2518 A>G *MCP-1* polymorphism. Although three genetic models were performed, the table only includes the comparison that shows significant differences. All children were divided into two groups, carriers and non-carriers of the A allele (AA+AG vs. GG). Interestingly, the G allele was the most frequent in children with insulin resistance (69%). However, we found that some A allele carriers also had insulin resistance (31%) (Table 2).

**Table 2:** Clinical and laboratory variables according to *MCP-1* genotypes

Variables		All children (n = 174)	AA+AG (n = 104)	GG (n = 70)	*P* value
Age (years)‡		9 (6-11)	9 (6-11)	9 (6-11)	0.39
Gender % (n)*					0.42
	Male	50.6 (88)	48.1 (50)	54.3 (38)	
	Female	49.4 (86)	51.9 (54)	45.7 (32)	
Weight (kg)‡		34.1 (20.1-59.2)	32.1 (20.3-59.1)	36.2 (20.1-64.9)	0.13
Height (cm)†		133.4 ± 11.5	132.6 ± 11.1	134.6 ± 12.2	0.26
BMI (kg/m^2^)‡		18.9 (14.3-28.2)	18.4 (14.3-25.9)	19.7 (14.5-29.1)	0.08
Obesity % (n)*					0.57
	No	51.1 (89)	53 (55)	49 (34)	
	Yes	48.9 (85)	47 (49)	51 (36)	
Waist circumference (cm)‡		67.5 (54-90)	67 (54-89)	70 (56-94)	0.10
Hip circumference (cm)‡		76 (62-96.5)	74 (62-96)	79 (62-96.5)	0.15
Waist-to-hip ratio‡		0.9 (0.8-0.97)	0.9 (0.8-0.97)	0.9 (0.8-0.97)	0.24
Arm circumference (cm)‡		21 (16-29)	21 (16.5-28)	22 (16-30)	0.20
Biceps skinfold (mm)†		15.6 ± 4.6	15.7 ± 4.9	15.5 ± 4.1	0.76
Triceps skinfold (mm)‡		15 (8.5-21.5)	15 (8.5-21)	15 (7.5-22)	0.97
Subscapular skinfold (mm)‡		13 (6-22.5)	12.8 (5.5-21.5)	13.5 (6-23)	0.65
Suprailiac skinfold (mm)†		18.2 ± 5.6	18.1 ± 5.5	18.2 ± 5.9	0.95
SBP (mmHg)‡		98 (80-115)	98 (80-117)	98 (81-110)	0.82
DBP (mmHg)‡		58 (49-71)	58 (49-72)	58.5 (50-70)	0.83
Hypertension % (n)*					0.47
	No	92.5 (161)	91.3 (95)	94.3 (66)	
	Yes	7.5 (13)	8.7 (9)	5.7 (4)	
Glucose (mg/dL)‡		96 (73-109)	96.5 (72-109)	95.5 (78-112)	0.71
Insulin (µU/mL)‡		6.9 (0.8-22.4)	6.6 (1.0-21.5)	8.2 (0.8-22.4)	0.17
HOMA-IR‡		1.7 (0.2-5.5)	1.5 (0.2-4.8)	1.9 (0.2-5.8)	0.15
Insulin resistance % (n)*					0.04
	No	67.2 (117)	73.1 (76)	58.6 (41)	
	Yes	32.8 (57)	26.9 (28)	41.4 (29)	
hsCRP (mg/L)‡		0.8 (0.1-6.5)	0.8 (0.1-8.0)	0.9 (0.1-6.3)	0.99
Leukocytes (10^3^/mm^3^)‡		7.6 (4.8-11.5)	7.7 (4.8-12.3)	7.6 (5.1-11.2)	0.64
Platelets (10^3^/mm^3^)‡		308 (219-412)	310.5 (224-413)	306 (205-410)	0.66

* Values are expressed as percentages and *n*. Chi-square test.

† Values are expressed as the means ± SD. Student's t test.

‡ Values are expressed as median (p5-p95). Mann-Whitney test. IR: insulin resistance; BMI: body mass index; SBP: systolic blood pressure; DBP: diastolic blood pressure; HOMA-IR: homeostasis model of assessment-insulin resistance; hsCRP: high sensitivity C-reactive protein.

The -2518 A>G *MCP-1* polymorphism was found in Hardy Weinberg Equilibrium in the total population (X^2^ = 0.57, P = 0.45), in cases (X^2^ = 1.03, P = 0.31) and in controls (X^2^ = 0.014, *P* = 0.91). The comparative analysis of genotype and allele frequencies between groups did not show significant differences; however, we found an association of the GG genotype with insulin resistance (OR = 2.2, 95% CI, 1.1-4.5; P = 0.03), determined by an adjusted genetic model (Table 3).

**Table 3:** Association of insulin resistance with genotype and allele frequencies of *MCP-1* polymorphism

Genotype/allele		Group without IR (n = 117) % (n)	Group with IR (n = 57) % (n)	*P*†	OR crude (95% CI); *P*	OR adjusted (95% CI); *P*‡
Frequencies				0.13		
	AA*	17 (20)	12 (7)		1	
	AG	48 (56)	37 (21)		1.1 (0.4-2.9); 0.89	
	GG	35 (41)	51 (29)		2.0 (0.8-5.4); 0.16	
Frequencies				0.08		
	A*	41 (96)	31 (35)		1	
	G	59 (138)	69 (79)		1.6 (1.0-2.6); 0.06	
Dominant model				0.41		
	AA*	17 (20)	12 (7)		1	1
	AG+GG	83 (97)	88 (50)		1.5 (0.6-3.7); 0.41	1.6 (0.6-4.3); 0.37
Recessive model				0.04		
	AA+AG*	65 (76)	49 (28)		1	
	GG	35 (41)	51 (29)		1.9 (1.0-3.6); 0.04	2.2 (1.1-4.5); 0.03
Additive model		-	-	-	1.5 (0.96-2.5); 0.07	1.7 (0.98-2.7); 0.05

* Reference category;

† Chi square test.

‡ Genetic model adjusted by age, gender, obesity and hypertension.

IR: insulin resistance; OR: odds ratio.

## DISCUSSION

In this study, an association between the -2518 A>G *MCP-1* polymorphism and insulin resistance is shown in a sample of Mexican children. Our results indicate that children with the GG genotype have a 2.2-fold higher risk of developing IR in comparison to those with the AA or AG genotype.

In contrast, a study in a German population found a low prevalence of insulin resistance and type 2 diabetes in carriers of the G allele compared with subjects homozygous for the A allele (24). In another study in a Chinese population, the G allele proved to be protective (adjusted OR=0.49, 95% CI, 0.32-0.77; P < 0.0001) against type 2 diabetes compared with subjects homozygous for allele A (31). These results suggest a protective role of the G allele for the development of insulin resistance and type 2 diabetes in the Chinese population. These differences may be attributed to the ancestry of the population; it is known that the Mexican population originated from mixed racial ancestry consisting of individuals from Europe (mainly Spain) or Africa who migrated to America where Native Americans of this region were living, giving origin to the Mexican mestizo population, who present a greater genetic diversity. This diversity can cause marked differences in allelic frequencies and patterns of linkage disequilibrium across their genome (32).

There are few studies reporting an association of -2518 A>G polymorphism with insulin resistance and type 2 diabetes, but other investigators have found associations of the GG genotype with high serum MCP-1 levels, hypertension, lupus nephritis and tuberculosis (27,33,34).

We found that the GG genotype was associated with an increased risk for developing IR when compared with children carrying other genotypes, which suggest that IR may also be related to increased serum *MCP-1* levels. In other studies, it was observed that insulin induces substantial expression and secretion of MCP-1, both *in vitro* in insulin-resistant 3T3-L1 adipocytes and *in vivo* in insulin-resistant obese mice; thus, MCP-1 was identified as an insulin-response gene (6). This result should be considered with caution, as it is necessary to perform a replication study in a larger sample to confirm these results.

Obesity and IR are characterized by chronic systemic low-grade inflammation. Markers of inflammation, such as tumor necrosis factor alpha (TNF-α), interleukin-6 (IL-6), CRP and MCP-1, are increased in peripheral blood levels in obesity and are associated with IR and may predict the development of type 2 diabetes (35). In this study, the children with IR showed increased hsCRP levels but not leukocyte or platelet counts. The GG genotype carriers did not present an increase in these markers, which could be an indicator of systemic inflammation not related to the development of insulin resistance in the studied children.

We observed that children with IR showed increases in all measures of central and peripheral adiposity compared to children without IR. Insulin resistance is a hallmark of obesity, emerging early in the metabolic syndrome, and is highly associated with increased visceral adipose tissue mass (36). Adipose tissue in obese subjects is characterized by macrophage infiltration, which is an early event contributing to the development of systemic insulin resistance. Indeed, transgenic mice that overexpress MCP-1 specifically in adipocytes develop adipose tissue inflammation and insulin resistance without obesity (37). This finding indicates that the subcutaneous adiposity may be an important predictor of IR in children.

Two main limitations should be considered in our investigation. First, the small sample size limited the statistical power. Second, MCP-1 levels were not measured, therefore the association of -2518 A>G polymorphism with MCP-1 levels remains uncertain in our population. Thus, future studies in Mexican children are necessary to determine MCP-1 levels.

In conclusion, the -2518 A>G *MCP-1* gene polymorphism may be related to the development of insulin resistance in Mexican children.
